# Biomechanical Stress in Obturator Prostheses: A Systematic Review of Finite Element Studies

**DOI:** 10.1155/2021/6419774

**Published:** 2021-08-16

**Authors:** Mohammed A. Mousa, Johari Yap Abdullah, Nafij B. Jamayet, Mohammad Khursheed Alam, Adam Husein

**Affiliations:** ^1^Prosthodontic Unit, School of Dental Sciences, Universiti Sains Malaysia, 16150 Kubang Kerian, Kelantan, Malaysia; ^2^Department of Prosthetic Dental Sciences, College of Dentistry, Jouf University, Sakakah, Jouf Province, Saudi Arabia; ^3^Craniofacial Imaging Laboratory, School of Dental Sciences, Universiti Sains Malaysia, 16150 Kubang Kerian, Kelantan, Malaysia; ^4^Division of Restorative Dentistry, International Medical University, Bukit Jalil, Jalan Jalil Perkasa-19, 57000 Kuala Lumpur, Malaysia; ^5^Department of Preventive Dentistry, College of Dentistry, Jouf University, Saudi Arabia

## Abstract

**Aim:**

This systematic review is aimed at investigating the biomechanical stress that develops in the maxillofacial prostheses (MFP) and supporting structures and methods to optimize it. *Design and Methods*. A literature survey was conducted for full-text English articles which used FEA to examine the stress developed in conventional and implant-assisted MFPs from January 2010 to December 2020.

**Results:**

87 articles were screened to get an update on the desired information. 74 were excluded based on a complete screening, and finally, 13 articles were recruited for complete reviewing. *Discussion*. The MFP is subjected to stress, which is reflected in the form of compressive and tensile strengths. The stress is mainly concentrated the resection line and around the apices of roots of teeth next to the defect. Diversity of designs and techniques were introduced to optimize the stress distribution, such as modification of the clasp design, using materials with different mechanical properties for dentures base and retainer, use of dental (DI) and/or zygomatic implants (ZI), and free flap reconstruction before prosthetic rehabilitation.

**Conclusion:**

Using ZI in the defective side of the dentulous maxillary defect and defective and nondefective side of the edentulous maxillary defect was found more advantageous, in terms of compression and tensile stress and retention, when compared with DI and free flap reconstruction.

## 1. Introduction

Management of patients who presents with such malignancy mostly necessitates surgical removal of a major portion of the palate with ablative surgery. The resultant paltal defect after surgery could be small or massive (when involves removal of a major portion of the palate, maxillary sinus, and/or nasal cavity). The patient's quality of life often collapses following the surgical resection of the tumor mass because of the corruption of function, speech, and aesthetics [1]. To overcomes the functional and psychological impact of the surgery, a surgical microvascular and/or prosthetic reconstruction must be carried out to improve the patient quality of life [2, 3]. Surgical rehabilitation of maxillary defects is not always possible due to the lack of donor sites, size of the defect, general health of the patient, and the risk of morbidity [4]. Maxillofacial prostheses (MFPs) are considered a cost-effective treatment option to reconstruct the lost dentition and missing structures in patients suffering from major maxillary defects [5, 6]. Even though many classifications have been introduced to distinguish the maxillary defect [7–10]; Aramany's classification was the most one followed by researchers due to its simplicity and smoothly communication among the maxillofacial prosthodontists [11].

When removable MFPs are used for rehabilitation of cases with major maxillary defects, the prostheses and their supporting structures are subjected to enormous pressure depending on the size and location of the defect, lack of adequate bone support, weight of the prostheses, the poor flexural strength, and low fatigue resistance of the prosthetic framework [12]. The developed pressure results in a concentration of the stress on the remaining part of supporting structures leading to bone resorption around the abutments and, eventually, failure of the prosthesis. Implant-assisted MFPs show many advantages comparing to the conventional ones, such as preservation of the remaining supporting structures, improvement of retention and stability, improvement of chewing efficiency, and improvement of patient's quality of life [13]. There is a direct relationship between success in dental treatment and biomechanics of materials used in dentistry [14]. The study of stress in prosthetic restorations has been reported before using different methods such as strain-gauge measurement, photoelastic stress analysis, and statistical finite element analysis (FEA) [15, 16]. FEA provides noninvasive reproducible qualitative and quantitative 2D and 3D information of biomechanical characteristics of dental prostheses and supporting structures with no need for ethical considerations when compared to other experimental methods [17–19]. FEA is carried out in three stages; the first stage is referred to as the "preprocessing stage" and it entails the creation of the FE model as well as specifying the properties of the materials. FE model can be generated in 3D by exporting the data from cone beam computerized tomography (CBCT) or magnetic resonance imaging (MRI), in (.stl) file format. Following the generation of FE model, the properties of the materials (and tissues) can be specified [20, 21]. The second stage is called "loading and boundary conditions" and it involves identifying the areas chosen to be the constrain and the area chosen to receive the load, and then the required load can be applied to the area of interest. The "postprocessing stage" which involves data analysis and result interpretation, is the third stage of FEA. Evaluation of biomechanical stress develps in different scenarios of MFPs has been done in literature, however, a systematic review of stress develops in the different scenarios MFPs, up to the authors' knowledge, has not yet been received in the dental literature. This was the purpose of this systematic review; to appraise the studies that used FEA to evaluate the biomechanical stress developed in removable MFPs and their supporting structures.

## 2. Study Design and Methods

This study was done after getting approval from the Human Research Ethics Committee of Universiti Sains Malaysia (HREC/USM) with JEPeM Code: USM/JEPeM/21030222 (Ph.D. proposal). The study followed the Preferred Reporting Items for Systematic Reviews and Meta-Analyses (PRISMA) guidlines. The questions of the research were formulated with the aid of the PICO format; while (P) is for the participants, (I) for the intervention, (C) for the comparison, and (O) for the outcome [22]. In scenarios with different forms of maxillary defects (P), what are the influences of maxillofacial prostheses (I) that fabricated with different designs and materials (C) on the distribution of stress and displacement of the prostheses during function (O)? An electronic search was conducted using the PubMed, Scopus, and Web of Science databases research tools. The inclusion criteria, as shown in Table 1, were the FEA studies, those conducted in English from January 2010 to December 2020, and only the studies that evaluated the stress on maxillary obturator prostheses. The exclusion criteria, as shown in Table 1, were *in vivo* studies, *in vitro* experimental studies, literature reviews, the articles that used FEA to estimate the stress in conventional fixed, removable partial, and complete denture, the articles that used methods other than FEA, letters to the editor, and unpublished data. The research was done by two authors (M.A.M and J.Y.A) independently conducted an electronic search on the 3 identified databases (PubMed, Scopus and Web of Science). The authors used the keywords “finite element analysis” and “obturators” for the preliminary survey. The articles were primarily screened by their title and abstract then by assessing the full text. In the selected articles, further research was performed in their references and citations for the possibility of including more articles. The quality assessment of the selected studies was not applicable as it is a mathematical way of estimating stress. The search was done to find answers to two questions. The first question was “where the stress distributed in the MFPs and supporting structures?”, while the second question was “What are the factors affecting the biomechanical stress distribution in the MFPs?”

## 3. Results

**Figure 1:**
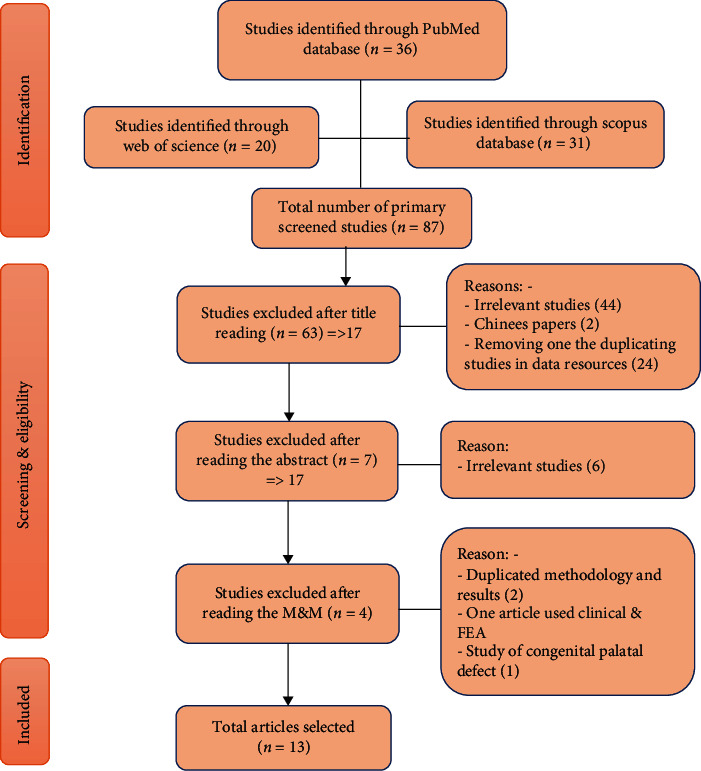
Flow chart for search process indicating numbers (*n*) of included and excluded studies.

During the nominated time of the study, 87 articles primarily were included in the survey. Out of these articles, 70 were excluded (based on initial screening of their titles and abstracts). Reasons for exclusion were either studies that were not related to the objectives of the current review, studies with duplicating results, or studies written in languages other than English. Four more studies were excluded after reading their methodology [23–26]. One of them evaluated the stress in congenitally unilateral palatal cleft scenario [25], one used mainly *in vivo* approach with no interpretation in the result and discussion sections regarding FEA [23], and four different studies were duplicated in their methodology and results [24, 26], so we chose the earlier studies [13, 27]. Finally, 13 articles were recruited for this review [13, 27–38]. The number of primary surveyed articles, number of excluded articles, reasons for exclusion, and the final recruited articles are shown in Figure 1.

Table 2 shows the summary of the studies that delivered on dentate maxillary defect scenarios, showing the type of research, the type of the maxillary defect examined, the design of the prostheses, the magnitude of the applied load, and assessment of the stress distribution in supporting tissues and the overlying prosthesis.

Table 3 shows the studies conducted to evaluate the stress developed in edentulous maxillary defects and their supporting structures. The results and findings were collected to identify the biomechanical stress developed in the maxillofacial prostheses and their supporting structures and how to manage it.

Except for three studies that followed Okay's classification [25, 31, 38], most of the recruited studies adhered to Aramany's classification [25, 31, 38]. Having read the methodology of the studies that used Okay's classification, we applied Aramany's equivalent design to their scenarios, to facilitate the comparison among the studies.  Table 4 shows the studies that examined the different scenarios of Aramany classification and the scenarios which failed to recieve attention in the literature.

The reviewed studies were aimed at identifying the maximum (tensile force) and minimum (compressive force) principle stress in the examined designs except for one study that assessed the differences in displacement between single- and two-piece closed hollow-bulb obturators [29]. In the reviewed studies, the stress was estimated in the prostheses for partially edentulous scenarios [13, 27, 29, 30, 32, 34–37], while in another it was examined in completely edentulous scenarios [25, 28, 31, 33, 38]. Six of the 13 studies reviewed in the current review, examined the influences of implant/s on the distribution of stress in the prostheses. Out of these six studies, only one was conducted in a dentate scenario [13], while the other five were for edentulous scenarios [25, 28, 31, 33, 38]. The used length and width of DI in all studies were the standard (4.1 − 4.5 × 10 mm) [28, 31, 33, 38], while ZI was 4 × 35 mm in all scenarios [13, 28, 33]. The stress distribution of single-piece hollow bulb section was examined by two studies [13, 25], while the two-piece was examined by two other studies as well [29, 36], and this mostly was to simulate the real situation.

There was vast differences in the magnitude of applied loads among the studies. The main reason for this is owing to the variety of the occlusal forces that can be found in the population, which are dependent on gender, age, general health, natural dentition, and anterior or posterior teeth. However, the mean maximum force 120-150 N were selected, either individually or collectively, in most of the reviewed studies, as it considers the mean maximum force for patients with remaining natural teeth and wearing removable obturator prostheses [13, 29, 30, 32, 35, 36].

## 4. Discussion

The purpose of this review was to compile all current information on the stress distribution developed in MFP and supporting structures, together with and the factors affecting it, from all FEA studies published within the last decade.

In the past decade, FEA has gained acceptance as a noninvasive and reliable method for simulating different dental defects and their corresponding prostheses, as well as analyzing the distribution of the stress within these prostheses and supporting structures [26]. However, the application of FEA has been used in limits in the identification of stress distribution in MFPs which may be due to the complexity of modeling the defects, simulation of the corresponding prosthesis, and the time involved.

Aramany's classification was the dominant classification that has been followed by most of the researchers. Essentially, this is mainly because of the simplicity of design and wide coverage of Aramany's classification which can be observed when applied to the other different classifications. Despite this, Aramany's classification overlooked the vertical extension of the maxillary defect, and thus, those authors who followed Aramany classification mistakenly assumed it does not exists [2].

There is a dependence between the stress developed in MFPs and magnitude, location, direction of the applied load, and number of remaining dentitions as well. As the load applied to the prosthesis does increase, the stress concentration in the prostheses and associated structures increases. This stress is shown as a compressive force, which concentrates at the resection line [31, 34]. Moeover, the stress developed within the prosthesis is increased as the number of remaining teeth decreases and as the size of the defect increases [32, 34]. The stress can be developed in either tensile or compressive form depending on where the load is applied. When the load is applied to the posterior portion of the prosthesis, the stress mainly developed in a compressive form and observed on the anterior midline. When the load applied on the anterior part of the prosthesis, the main stress becomes in the form of tensile and observed throughout the midline region [34].

In the studies that evaluated dentulous scenarios of Aramany's class I and class II palatal defect, they found that the maximum stress concentration is located around the cervical half of the roots of the teeth next to the resection, central, and lateral incisors of the contralateral side in class I or canine in class II [13, 30, 35]. The cobalt-chromium alloy was found to produce more stress on the remaining teeth, when used as a major connector and retainers, compared to titanium alloy which showed more flexibility. Desbite this, due to titanium decreased rigidity, the major connector is deflected toward the areas where the force is applied [30, 35]. The use of Vertex polymer as a retainer with an occlusal plate on the main abutment teeth was found to reduce stress on the teeth without compromising the stability of the prosthesis [24]. There were no significant differences in the distribution of stress between the single- and two-piece hollow obturators, although the two-piece obturator showed a slight lower stress value than the single-piece obturator [29, 36].

Free flap reconstruction of unilateral dentate maxillary defect is used, as a surgical method, to simplify the prosthetic rehabilitation. The greates stress concentration, however, was found at the junction of the flap and the palatal bone under obturator. This stress was found four times more than the stress developed under traditional obturator, thus results in rapid loss of bone support at the junction between the flap and the palate, causing instability of the obturator [37]. Another way to decrease the stress concentration in a dentate Aramany's class I was to assist and retain the MFP with ZI/s, since the DI cannot be used in the defective side. When one or two ZI are added to assist the MFP, the ZI shares the stress with the abutment teeth on the contralateral side, thereby reducing the torque on the abutment teeth and reducing the rotation of the prosthesis toward the defect [13].

The design of MFPs in dentate scenarios receiving the least attention in literature is Aramany's class III. To the best of authors' knowledge, there was a lack in literature, up to the authors' knowledge, of this design from the perspective of FEA. This may be because Aramany's class III might resemble, to an extensive degree, the conventional Kennedy class III. In contrast, Aramany's class I and IV were the two designs which garnered the most attention [13, 27, 29, 30, 32, 36]. Owing to the extreme defect associated with these scenarios, which have a substantial impact on the biomechanical stress in the corresponding prostheses and their supporting structures, might prompted the researchers to investigate them. The massive bone defect makes the MFPs tend to rotate toward the defect around the midline of the remaining part of the maxilla, which affects the stability and periodontium of the remaining abutment teeth [34]. It was possible to reduce the stress in Aramany's class IV by modifying the clasp design [32]. Researchers found that the multiple roach clasps reduce the stress on the MFP supporting structure when compared with multiple Aker's clasps [32]. There was a lack of literature, up to our knowledge, about the influences of assisting the MFP in Aramany's class IV with ZI.

In contrast to the lack of coverage to some of Aramany's classification defects in dentate scenarios, there has been wide literature coverage of the various types of edentulous Aramany classification over the last decade [28, 31, 33, 38]. In the study done by de Sousa and Mattos, they follow Okay's classification Ia, II, and III maxillary defects, which are equivalent to Aramany's class II, I, and IV, respectively [31]. Same as in dentate maxillary defects, the displacement of the MFP at the resection line increases as the defect increases, reaching a maximum in Okay's class III (Aramany's class IV) [31]. Even though adding DI on the nondefective side was found to decrease the stress on the remainder of supporting structure, the implant/s are still subjected to high compressive stress especially when the defect becomes massive as in Okay's class III situations. The stress in Okay's class III is tensile in its dominant form and usually concentrated around the cervical part of the cortical bone around DI, which may jeopardize the survival of the implants. One method to decrease the stress on the DI on the nondefective side is adding one (or two) ZI on the defective side [28], or adding one ZI on each side, which was found beneficial in reducing the stress comparing adding two or three DI in the nondefective side [33].

The amount of stress concentration depends not only on the type and number of the implants but also on the type of prosthetic materials, the type of the connectors, and the abutments of implants. Although the polyetheretherketone (PEEK) showed the least stress on the residual ridge (bone), it showed the highest stress concentration in the prosthetic screws and clips. In contrast to PEEK, Co-Cr showed the highest stress on the bone but the lowest stress on the prosthetic screws. The supporting bone and prosthetic screws showed adequate stress concentration with titanium framework [38].

There is no disputation, that forces of occlusal and masticatory function are completely transmitted to restoration and supporting structure without loss [39, 40]. As per the reviewed studies, these forces could be concentrated in certain portions in the prostheses and their supporting structure, leading to permanent deformation (or even fracture to these portions) and/or tissue resorption to the supporting structure. From the authors' point of view, the key factor for the success or failure of MFPs is to evaluate the pattern in which stresses are transferred to the supporting structures to minimize it.

This systematic review showed the deficiency in the literature regarding different designs of the different scenarios of maxillary defects and their corresponding prostheses. Further researches are needed to examine the overlooked scenarios of maxillary defects as shown in this review.

This review focused only on the FEA studies to identify the stress concentration in MFPs and the methods to reduce this stress. Despite its noninvasive and flexible nature, FEA has many inherited limitations when comes to simulating maxillary defect and their corresponding prostheses. Some, but not all, of these limitations include the homogeneity of the used materials (ignoring the manufactures and laboratory errors during processing), isotropic linearity (assuming the material when respond to load, shows the same reaction in all direction), biology of the tissues (assuming the bone showing the same density in all areas), osseointegration (assuming 100% osseointegration), and the other physiologic differences that exist among the patients. Clinical validation using methods, such as photoelastic stress analysis and strain gauge analysis, would be crucial to validate the FEA.

## 5. Conclusion

Within the limitation of this review, we can conclude the following

1. The stress and displacement of MFPs are highly influenced by the sizes and characters of maxillary defects, availability of adequate undercuts, and health and position of the remaining dentitions
2. The stress is mainly concentrated on the resection side and the apices of the teeth next to the defect
3. Using DI in the nondefective of maxillary defect reduces the stress on the supporting structure as the implant share the stress with the abutments
4. Adding ZI in the defective side of dentulous and in the defective and nondefective sides in edentulous maxillary defect is considered a key factor in reducing the displacement of maxillofacial prostheses. It may also decrease the need to DI, the need to use clasps on the teeth next to the resection, and - to massive surgical free flap reconstruction

## Figures and Tables

**Table 1:** The inclusion and exclusion criteria of the present study.

Inclusion criteria	Exclusion criteria
(1) The studies conducted between January 2010 and December 2020 (2) Studies conducted in the English language (3) *In vitro* mathematical studies (4) Studies conducted only on partial and complete removable maxillofacial obturators (5) The case with surgical maxillary defect (6) Studies used only FE methods to predict the distribution of the stress and displacement in MFP	(1) The studies conducted out of the inclusion time range (2) Studies conducted in a language other than English (3) *In vivo* and *in vitro* lab experimental studies (4) Studies conducted on conventional partial and complete removable dentures (5) Studies conducted on fixed prostheses (6) The scenarios with congenital maxillary defect (7) Studies conducted on partial MFP with complete acrylic base (8) Studies that used other than FE method to predict the stress

**Table 2:** Summary of the studies estimated the distribution of the load in obturators in dentulous scenarios.

Authors and year	Type of maxillary defect	Type of prosthesis	Design of the prosthesis	Length and width of implant, if used	Type of abutment if implant used	Magnitude and direction of the applied load	The main outcome
Sudhan et al. 2020 [36]	Aramany's class I maxillectomy defect	Metal-acrylic partial MFP	Using metal base RP with single- and two-pieces hollow bulbs in the defect side and tripodal design of the occlusal rests and clasps on central, premolars, and molars in the unaffected side	NU	NA	VLF 150, 200, and 250 N were applied on the incisal and occlusal surfaces of anterior and posterior teeth	(i) No SD shown between one piece and two pieces hollow bulb obturator regarding the stress concentration (ii) The highest stress concentration was concentrated in the lateral wall of the defect

Shah et al. 2019 [35]	Aramany's class II defect	Removable with two types of metal (Co-Cr and Ti alloy)	Complete palate major connector with no bulb portion in the defect side, with embrasure clasp and occlusal rests on first molar and second molar, and cingulum rest on canine	NU	NA	120 N was applied vertically and horizontally on the prosthesis (the teeth not identified)	(i) The Co-Cr showed maximum stress when compared with titanium alloy (ii) The deflection is more in Ti when compared with Co-Cr (iii) The abutment teeth showed the highest stress in Co-Cr when compared with Ti alloy

Arabbi et al. 2019 [30]	Aramany's class I defect	Removable with two types of metal (Co-Cr and Ti alloy)	Complete palate major connector, with no bulb portion. cingulum rest on the canine, occlusal rests between I premolars and I molar, embrasure clasp between the II premolar and the I molar, and I Bar retainer at the central incisor.	NU	NA	120 N was applied vertically and horizontally on the prosthesis (the teeth not identified)	(i) The Co-Cr showed maximum stress when compared with titanium alloy (ii) The deflection is more in Ti when compared with Co-Cr, especially upon the horizontal direction

Anitha et al. 2019 [29]	Aramany's class I	Metal-acrylic	Using metal base RP with single- and two-piece hollow bulbs in the defect side and tripodal design RPD. Two pieces obturator used Co-Sm magnet for retention	NU	NA	Three VLF 150 N, 200 N, and 250 N were applied to the anterior and posterior teeth (the teeth not identified)	(i) As the magnitude of the force increases, the amount of deflection of the prostheses increases. It happened slightly more in two pieces compared to one piece (ii) It is mainly concentrated in the premolar area compared to the anterior and posterior
Shulatnikova et al. 2016 [27]	Aramany's class I	Prosthesis fabricated from polymer material Vertex ThermoSens with the addition of nanostructured TiO2	Full palatal coverage with and without occlusal plate (extension on the occlusal surface of the abutment teeth)	NU	NA	Total 720 N applied to the occlusal plate when used or the abutment tooth if not used	Vertex polymer with occlusal plate leads to reduction of stress on the abutment without adverse effect on the stability of the prosthesis
Wang et al. 2013 [13]	Aramany's class I	Metal-acrylic	Full metal in the intact part and acrylic in the defective part. The design of the metal is tripodal.	ZI 4.5 in diameter. Length not identified	Type of attachment not mentioned	VLF 150 LLF 150 with angle 26.5° to vertical	(i) The maximum stress was shown in the conventional when compared with one and two Z. implant models (ii) The highest stresses were arising at metal-line palate plate and clasps (iii) Z. implants share the stress with teeth (iv) The distal implant was found to receive higher stress than the mesial implant
Hase et al. 2014 [32]	Aramany's class IV	Co-Cr base	Full metal with NO bulb section. Double Akers clasps and multiple roach clasps were used in remaining abutment teeth for comparison	NU	NA	120 VLF and 90 oblique LF N were applied in equal distribution on the premolar and molar teeth.	Both types of clasps produce stress in the abutment teeth. However, more stress concentration was observed around the premolars upon using double Aker clasps when compared to the stress that arises in the buccal and distal part of the second molar when using multiple roach clasp
Miyashita et al. 2012 [34]	Aramany's class IV	Metal and acrylic	Full coverage major connector with no bulb section linear design including two embrasure clasps between 1^st^ and 2nd premolars and 1^st^ and 2^nd^ molar teeth.	NU	NA	120 N applied on two points, one on anterior and one on posterior teeth (the direction of load not identified)	(i) The prosthesis showed a tendency to rotate under posterior load. The axis of rotation was located near the resection line at the midline of the maxilla (ii) Compressive stress was observed on the surface of the palate, distributed to the alveolar ridge, and on the resection line (iii) The intensity of this stress was not sufficient to cause bone resorption

Sun and Jiao 2010 [37]	Aramany's class I with free flap construction	Cast metal base partial denture	As the prosthesis was fabricated after reconstruction it looked like the conventional metal prosthesis. The designs included cast palatal base with 4 circumferential clasps	NU	NA	450 N VLF was applied as follows: (i) 150 N for artificial teeth distributed as 10 N on every anterior tooth, 20 N on every premolar, and 40 N on every molar (ii) 300 N applied on the contralateral side distributed as double as artificial teeth	Most of the stress was concentrated at the junction line between the palatal and the free flap and anterior teeth.

VLF: vertical loading forces; HLF: horizontal loading forces; MFP: maxillofacial prosthesis; NU: not used; NA: not applicable; ZI: zygomatic implant; DI: dental implant; SD: significant differences; Co-Cr: cobalt chromium; Co-Sm: cobalt samarium; Ti: titanium; TIO_2_: titanium dioxide; PEEK: polyetheretherketone.

**Table 3:** Summary of the studies estimated the distribution of the load in obturators in edentulous scenarios.

Authors and year	Type of maxillary defect	Type of prosthesis	Design of the prosthesis	The length and width of the implant, if used	Type of abutment if implant used	Magnitude and direction of the applied load	The main outcome
Villefort et al. 2020 [38]	Type Okay's class IIb (equivalent to Aramany's class III)	The prostheses are composed of 3 different materials including PEEK, Ti, and Co-Cr	The different prostheses are supported with 5 implants and connected with a milled bar and clips system	DI 4.1 × 10 mm	External hexagonal dental implant	100 N on the cingulum of anterior teeth and 150 to the 1^st^ molar	(i) PEEK showed less stress in the bone tissue and improve the integrity of the bar and bar-clip attachments. However, it increases the risk of failure of prosthetic screw (ii) Co-Cr decreases the stress on the prosthetic screw, but it increases it on the bone (iii) Ti shows adequate behaviour regarding the stress on bone and integrity of the prosthetic screw
Akay and Yaluğ 2015 [28]	Aramany's class IV	Acrylic denture with no bulb section	Three different designs were modeled: 1 ZI in the defective side and 1 DI in the nondefected side, 1 ZI in the defected side and 2 DI in the nondefected side, and lastly 2 ZI, one on each side.	DI with 4.5 × 10 mm and ZI with 4 × 35 mm	Locator attachment used for all implants	150 N VLF was applied on each part alone then on both sides at the same time	(i) Using ZI in the nondefected side decreases the stress when compared with using 2 DI with locator attachment
de Sousa and Mattos 2014 [31]	Okay class Ia (equivalent for Aramany class II), II (equivalent to Aramany class I), and III (equivalent to Aramany's class IV)	Acrylic denture with NO bulb section	3D models were simulated as the following designs: for Okay class Ib, 6 implants were inserted in the canine and lateral incisors on both sides and in the left first premolar and molar regions. For Okay class II, 4 implants were positioned in the left lateral incisor, canine, first premolar, and molar regions. For Okay class III, 2 implants were placed in the left first premolar and molar regions	11.5 mm length (the width not mentioned)	Using bar and clips retention system (the thickness and height of the bar not mentioned)	80 N VLF was applied on the occlusal surface of posterior teeth and 35 N VLF was applied on the anterior teeth	(i) Dislodgement of the prostheses was clear at the resection line (ii) The dislodgment increases as the defect increase, reaching the maximum in Okay class III (Aramany class IV) (iii) Adding implants in both sides decrease the displacement of the prostheses
Korkomaz et al. 2012 [33]	Aramany's class I	Acrylic implant assisted overdenture	4 prostheses were simulated as the following designs: model 1; with 2 ZI (I on each side) and 1 DI (in the anterior part). Model 2; 2 ZI and 2 DI. Model 3; 2 ZI and 3 DI, and model 4; 1 ZI and 3 DI. When 2 ZI is used, they placed one on each side DI placed on the nondefected side	DI 4.1 × 10 mm ZI 4 × 35 mm	Titanium U-shaped Dolder bar with a height of 3 mm was modeled in all models generated.	150 VLF were applied on 15 points in form of 10 N on each one. The points distributed between the two premolars and 1^st^ molar	(i) Use of ZI on the nondefected surface provided decreased stress values around implants (ii) Increasing the number of DI on the nondefected side did not have any additional advantage regarding to stress value

MFP: maxillofacial prosthesis; NU: not used: NA: not applicable; ZI: zygomatic implant; DI: dental implant; Co-Cr: cobalt chromium; Co-Sm: cobalt samarium; Ti: titanium; TIO_2_: titanium dioxide; DI: dental implant; ZI: zygomatic implant; PEEK: polyetheretherketone.

**Table 4:** Summary of the selection process of the review.

The maxillary defect	The type of the scenario	The study	Aims	
			Stress	Displacement
Aramany's class I	Dentulous	Sudhan et al. 2020 [36], Arabbi et al. 2019 [30], Anitha et al. 2019 [29], Shulatnikova et al. 2016 [27], Wang et al. 2013 [13], and Sun and Jiao 2010 [37]	√	√
	Edentulous	de Sousa and Mattos 2014 [31] and Korkmaz et al. 2012 [33]	√	√

Aramany's class II	Dentulous	Shah et al. 2019 [35]	√	√
	Edentulous	de Sousa and Mattos 2014 [31]	√	√

Aramany's class III	Dentulous	—	—	—
	Edentulous	Villefort et al. 2020 [38] and de Sousa and Mattos et al. 2014 [31]	√	√

Aramany's class IV	Dentulous	Hase et al. 2014 [32] and Miyashita et al. 2012 [34]	√	√
	Edentulous	Akay and Yaluğ 2015 [28] and de Sousa and Mattos 2014 [31]	√	√

Aramany's class V	Dentulous	—	—	—
	Edentulous	—	—	—

Aramany's class VI	Dentulous	—	—	—
	Edentulous	—	—	—

## Data Availability

All data are available within the manuscript.
